# Optimization of salinity and composition of injected low salinity water into sandstone reservoirs with minimum scale deposition

**DOI:** 10.1038/s41598-023-40067-y

**Published:** 2023-08-10

**Authors:** Masoud Bijani, Ehsan Khamehchi, Mehdi Shabani

**Affiliations:** 1 Department of Petroleum Engineering, Amirkabir University of Technology (Tehran Polytechnic), Tehran, Iran; 2 Regional Technical Advisor at STRATUM Reservoir, Middle East, Abu Dhabi, UAE

**Keywords:** Geology, Hydrology, Chemistry, Energy science and technology, Engineering

## Abstract

In this study, a mechanistic and comprehensive examination of the impact of the scale formation situation of different diluted seawater levels was conducted to investigate the influence of important factors on the performance and efficiency of low salinity water. To clarify the effective participating mechanisms, scale precipitation by compatibility test, field emission scanning electron microscopy (FESEM) and energy dispersive X-ray spectroscopy (EDX) analysis, zeta potentials as surface charge, ion concentration changes, contact angle, pH, CO_2_ concentration, electrical conductivity, and ionic strength were analyzed. The results showed that increasing the dilution time to the optimal level (10 times-diluted seawater (SW#10D)) could effectively reduce the amount of severe precipitation of calcium carbonate (CaCO_3_) and calcium sulfate (CaSO_4_) scales. However, the reduction in CaCO_3_ scale precipitation (due to mixing different time diluted seawater with formation brine) and its effect on the wettability alteration (due to the change in surface charge of OLSW/oil and sandstone/OLSW) had higher impacts. The zeta potential results have shown that OLSW with optimum salinity, dilution, and ionic composition compared to different low salinity water compositions could change the surface charge of OLSW/oil/rock (− 16.7 mV) and OLSW/rock (− 10.5 mV) interfaces toward an extra negatively charged. FESEM and contact angle findings confirmed zeta potential results, i.e. OLSW was able to make sandstone surface more negative with diluting seawater and wettability changes from oil-wet toward water-wet. As a result, SW#10D was characterized by minimum scaling tendency and scale deposition (60 mg/l), maximum surface charge of OLSW/oil/rock (− 16.7 mV), and the potential of incremental oil recovery due to wettability alteration toward more water-wetness (the oil/rock contact angle ~ 50.13°) compared with other diluted seawater levels.

## Introduction

Fossil fuels are a significant portion of the worldwide energy supply^1^. One of the oldest and well-known methods used in oil reservoirs for pressure maintenance and improved oil recovery is water injection. Water injection is the most considerably applied increased oil recovery method after the natural recovery of oil reservoirs^2^. Lately, low salinity water injection (LSWI) and smart water injection (SWI) showed positive effects in the recovery factor of oil reservoirs^3^. Various laboratory experiments and field applications have shown increasing oil production because of modification of ion amount or reducing salinity level of seawater and increasing the dilution ratio of seawater^4–11^. LSWI and SWI can be applied as improved oil production methods after changing the wettability of oil reservoirs^12–16^. The prominent mechanisms of LSW flooding were wettability alteration, fine migration, rock dissolution, multiple ion exchange (MIE), and double layer expansion^17,18^. Though, the dominant mechanism is referenced as wettability alteration in literature^14,18^. Smart water as a kind of injection water can have two meanings under different definitions as follows^19–21^:

- Application of saline water at low concentrations, neglecting the type of ions.
- Synthesis of a new water composition according to ion design considering their concentration and type.

In this method, the amount of salinity decreases from about 1000 to 7000 ppm. Also, the potential determinant ions (PDI) of calcium (Ca^2+^), magnesium (Mg^2+^), and sulfate (SO_4_^2−^) in injection water change the wettability of rock towards higher hydrophilicity and increase oil recovery^14,22–26^. Divalent ions include calcium (Ca^2+^), magnesium (Mg^2+^), and sulfate (SO_4_^2−^) activate surface charges. Also their concentrations in the solution determine the polarity and surface charge density of the rock and affects the reaction between oil and the rock surface^27^. According to results obtained from the tertiary water injection method in oil reservoirs, 18% incremental oil recovery was obtained by diluted seawater in a stepwise manner, 2, 10, and 20-times diluted seawater. Subsequently, the two leading causes during smart water flooding can modify the wetting characteristics of sandstone and carbonate rocks as wettability alteration as follow^12,14,28,29^:

1. Increased divalent ion concentrations (including Ca^2+^, Mg^2+^, and SO_4_^2−^) in the injected seawater.
2. Reduced salinity of the injected brine.

Thus, according to previous studies that modifying the composition of injected brine can result in more oil produced from oil reservoirs. Injection brine composition is a more important factor in scale deposition because scale formation happens when the formation water is mixed with other incompatible brines such as injected waters^30^. Formation water has high-level salinity, total dissolved solids (TDS), total suspended solids (TSS), and different anions and cations in solution^31^. It indicates that the composition of the injected seawater is a significant quantitative parameter also the injected seawater quality should control similarly. It is important to examine the compatibility of smart water and low salinity water with reservoir conditions to prevent the scale formation^32^. Briefly, the possibility of the mineral scale formation as formation damage in oil reservoirs during smart water and low salinity water is important because of incompatible waters when mixing the brine injection with the formation water. To date, various studies have been conducted to evaluate the incompatibility of formation water and injection water^33^. Two major types of inorganic scales that are usually formed in oil reservoirs during water flooding operations are sulfate and carbonate scales^34,35^. One of the main causes of carbonate scales is usually the incompatibility of formation and injection waters mixing with different ratios of calcium and bicarbonate-rich water like seawater mixed with formation water. It can precipitate calcium carbonate and iron carbonate^35,36^. Calcium carbonate (CaCO_3_) as an inorganic scale in calcite form can contribute to difficult oil industry problems. Therefore, scale formation inhibition is more useful than the removal of scales and the use of inhibitors in different chemicals and solvents. These operations are economically costly and contribute to environmental problems^37^. Predicting the potential scaling is one of the most effective methods for controlling the risk of problems such as scale formation during water flooding operations^38^. This would be pretty helpful for handling mineral scale challenges and developing the most effective technique for inhibiting the formation of scales in oil fields^36^. The present study was conducted to explore the most critical challenges of water-flooding process associated with the potential of mineral scale deposition like CaCO_3_ during the experiment of low-salinity water flooding in a sandstone reservoir. Few researches have been done on accurate investigation of important factors on scale precipitation of injected brines during low salinity water injection for EOR in oil reservoirs. Unlike previous experimental works^3,12,13,39–45^, the present study set to extend the investigation of scale formation in different low salinity water compositions by different effective parameters together. These parameters include the effect of salinity, dilution ratios of seawater, electrical conductivity (EC), ionic strength (IS) of brines, type and concentration of ions and salts, pH, and surface charge in aqueous solution on scale formation and wettability alteration.

The objectives of the study were as following:

1. Examining the effects of ions (Ca^2+^, HCO_3_^−^, and Mg^2+^), the salinity of injection brines, physicochemical parameters such as pH, EC, IS, CO_2_ concentration as solution in brine. In addition, dilution times of seawater on the amount of CaCO_3_ precipitation from mixing seawater, diluted seawater levels, and formation water with Ca^2+^ ions were investigated.
2. Optimizing the ion contents and diluted seawater levels as low salinity water to improve wettability alteration and reduce the formation damage.
3. Evaluating the effect of optimum LSW composition with minimum scale precipitation on wettability alteration.
4. Assessing the compatibility of thermodynamic data obtained from the mixed brines using OLI ScaleChem and comparing the collected data and experimental results.

**Figure 1:**
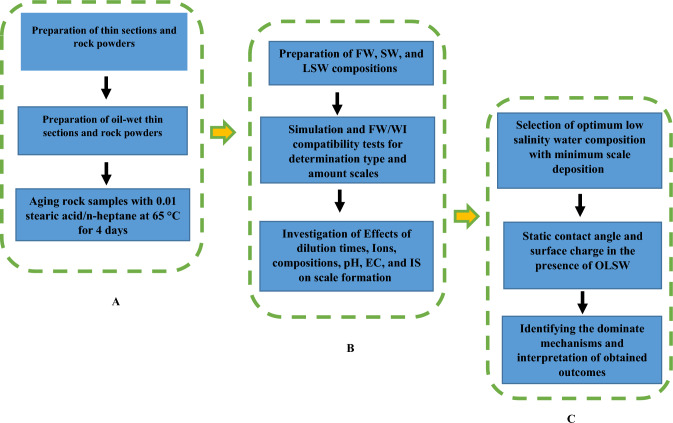
Schematic diagram of the experiment and simulation steps.

An analysis of effective parameters was conducted in this research. To achieve objective 1, we investigated the type and amount of scale deposition by water compatibility experiments, pH, ionic strength, electrical conductivity measurements, and FESEM-EDX experiments. To achieve objectives 2 and 3, the effect of different low salinity water compositions and optimum low salinity water on wettability alteration were investigated by contact angle tests, FESEM, and the brine/rock and brine/oil surfaces charges by zeta potential experiments. Finally, to achieve objective 4, OLI ScaleChem software, brine compatibility experiments, and FESEM-EDX experiments were used to match experimental data and simulation also the results were compared together. Figure 1 shows a schematic diagram of the simulation and experiment steps for all scenarios. While Fig. 1A shows steps of preparation of thin sections and rock powders and after aging rock samples process in the reservoir conditions. Also, Fig. 1B shows steps of investigation of important factors on scale formation during comptability experiments of injected brines with formation water. Finally, Fig. 1C presents steps of selection of optimum low salinity water composition and identifying the dominate mechanisms.

This research opens new horizons in compatibility experiments for optimization of dilution ratio and ion composition of LSW injection technique for EOR with minimum scale risk and high wettability change performance, which has not been investigated before. Also, this research has the novel points as following: (1) unlike the already experimental studies, the study presents a comprehensive and simultaneous physiochemical understanding (impact of ions, pH, surface charges, salinity, ionic strength, electrical conductivity, and dilution ratios) to elevate potential scale formation during low salinity water utilization to improve wettability alteration in sandstone oil reservoirs; (2) comprehensive and simultaneous investigation for determining the optimum dilution ratio, salinity, LSW composition according to minimum scale-risk; (3) this study illustrated that SW#10D with optimum salinity, dilution, and ionic composition was prepared for low salinity water injection, which could improve wettability alteration to water-wet state. However, increasing and reducing dilution times of SW#10D can enhance the risk of scale precipitation and increase cost and operation at the field scale. As a result, OLSW (SW#10D) is characterized by the control potential for scale deposition and more capacity for wettability alteration at the same time. In the final, this work presents the result of a comprehensive examination involving the developed LSW with an optimum concentration as a new promising technique for EOR that could be more efficient and cost-effective for successful wettability alteration and formation damage in minimum scale deposition.

## Prediction of scale formation in oilfields

One of the significant challenges facing oil industry is predicting mineral scale formation because of several factors such as supersaturation, changes in the thermodynamic condition, ionic strength, pH, alkalinity, and flow velocity^36,46^. There are many different solubility prediction methods for inorganic scales in oil and gas wells and reservoirs. Thermodynamic models are the most effective method that can be applied to inhabit and control mineral scale formation in wells and reservoirs. Because these models included the interference and effect of important thermodynamic factors such as temperature and pressure. According to the solubility limitations, available thermodynamic models can only predict the scale deposition's potential^47,48^. The supersaturation of the aqueous solution can contribute to the nucleation and growth of the scales, as demonstrated by the saturation index (SI) and the saturation ratio (SR)^33,36,39^.

### Prediction of saturation index (SI)

To evaluate of water compatibility between the LSW and formation brine, SI is regard a measurable parameter to determination the scale deposition^49^. SI is expressed as scale precipitation of brine ratio to scale deposition, and the logarithm of ion concentration products must be divided by an equilibrium constant. For each scale, SI is defined as follows^36,50,51^:

1 $$ SI = \left( {\frac{ion\,activity\,product}{{K_{sp(P,T,I)} }}} \right) $$

Or

2 $$ SI = \log (SR) = \log \frac{[Me] \times [An]}{{K_{sp(P,T,I)} }} = \log [[Me] \times [An]] - \log K_{sp(P,T,I)} $$

Hence, based on the ionic activity coefficient, Eq. (5) can be written as the following formula^36^:

3 $$ SI = \log (SR) = \log_{10} \left\{ {\frac{{\alpha_{{_{Me} }} \times \alpha_{{_{An} }} }}{{K_{{_{sp(P,T,I)} }} }}} \right\} = \log \{ \alpha_{{_{Me} }} \times \alpha_{{_{An} }} \} - \log K_{{_{sp(P,T,I)} }} $$

While in these equations [Me] include cations of Mg^2+^, Ca^2+^, Sr^2+^, and Ba^2+^, [An] includes anions or negative ions such as HCO_3_^−^ and SO_4_^2−^, and K_sp_ denotes the solubility product of aqueous solution under reaction situation. If the saturation index equals 0 (SI = 0), then a liquid medium is saturated. In addition, when the saturation index has lower values than zero (SI < 0), the undersaturated situation and scale will not precipitate^52^. Ultimately, when the SI has greater values than 0 (SI > 0), it indicates that an aqueous solution is supersaturated and mineral scale tends to form^35,36,47,53,54^. The SI values for an aqueous solution under different conditions are shown in Table 1^36,55^.

**Table 1:** Interpretation of saturation index.

SI	Description
SI = 0	An aqueous solution is saturated, indicating that the scale has no tendency for the formation or dissolution
SI < 0	An aqueous solution is undersaturated, suggesting that scale has no tendency for dissolution
SI > 0	An aqueous solution is supersaturated; resulting scale has tendency for formation

## Materials and methods

One critical parameter to study the deposition–precipitation mechanism is the composition of formation and injection brines to elevate scale formation phenomena and the mixing ratios of two brines. This study was conducted on low salinity water obtained from the Persian Gulf (seawater) for feasibility as injection water and formation damage created as mineral scale formation. For the investigation of the value and type of scales, the compatibility brine tests of mixing low salinity water compositions with formation water were performed at different ratios of 20%, 40%, 50%, 60%, and 80% and reservoir temperature (T = 65 °C). As a result, the maximum scale precipitation ratio was obtained to concern mineral scale formation. In the second stage, The FESEM-EDX tests were conducted on filters samples after compatibility tests for determination of scale types. In the third stage, The FESEM image and contact angle test were conducted to evaluate the wettability alteration. In the last stage, the lab tests were carried out on sandstone rock powder for the Zp evolution and effect surface charges on scale deposition and wettability alteration in line with the FESEM and contact angle findings.

### Materials

#### Brines

In the first step, formation and injection brines were synthesized, whereas formation water had the same composition as the reservoir brine, and Persian Gulf water (PGW) (seawater that is nearby to the sandstone field from southwest Iran) was chosen as a suitable supply for injection water. All samples of the brine compositions were synthesized in the lab that could dissolve in the given quantity of six salts at high purity from Merck Chemicals (purity of 99.5%), including NaCl, KCl, Na_2_SO_4_, NaHCO_3_, MgCl·6H_2_O, and CaCl_2_·2H_2_O dissolved in deionized water. deionized water as 3-time evaporation distilled water with a resistivity of 18.2 MΩ cm was utilized. Table 2 shows the compositions of the formation and injection brines (seawater) used in this study. The formation water composition belongs to one of the oil reservoirs in southwest Iran. Also, the water composition of the Persian Gulf was used as injection water (seawater). The total dissolved solids (TDS) were 195,671.03 and 40,687 ppm for FW and SW, respectively. It should be mentioned that formation water had high-level salinity, total dissolved solids (TDS), total suspended solids (TSS), and different anions and cations in solution^31^. Various concentration levels of the injection water were prepared based on seawater collected from the Persian Gulf composition.

**Table 2:** Complete water compositions of the formation water and seawater**.**

Ions	Unit	Formation water	Seawater (Persian Gulf)
Na^+^	mg/l	59,142.47	126.53
K^+^	mg/l	0	420
Ca^2+^	mg/l	135,00	498
Mg^2+^	mg/l	1,725	1408
SO_4_^2−^	mg/l	449	3037
Cl^−^	mg/l	120,444.44	22,598
HCO_3_^−^	mg/l	293	73
TDS	mg/l	195,671.03	40,687
Density	g/ml	1.126	1.02
Total alkalinity	mg/l as HCO_3_	293.06	73
Salinity	mg/l as NaCl	198,131.10	37,173.71
pH	–	6.5	8.13
EC	ms/cm at 25 °C	130	58
Ionic strength	Molal	1.73	0.829

#### Crude oil

The crude oil from one of the Iranian southwestern reservoirs was applied in this work. The chemical composition and physical properties of the crude oil is presented in Tables 3 and 4. Also, Table 3 states that the value of C_1_ is equal to 45.59% and C_7+_ is equal to 30.24%.

**Table 3:** Crude oil properties.

Components	C_1_	C_2_	C_3_	iC_4_	nC_4_	iC_5_	nC_5_	C_6_	C_7_	C_8_	C_9_	C_10_	C_11_	C_12_^+^	H_2_S	CO_2_
Reservoir Oil (mol%)	45.59	7.02	4.28	0.87	2.13	0.88	0.88	4.78	1.33	3.14	1.72	2.04	1.78	23.21	0	0.21

**Table 4:** The physical properties of crude oil.

M.W (g/mol)	Sp.gr	API	Viscosity (cp)
86	0.64	32.24	0.41

#### Stearic acid and n-heptane chemicals

A concentration of 0.01 molar of stearic acid solution in n- heptane was applied to wettability alteration the rock samples. The n-heptane and stearic acid were purchased from Merck.

#### Sandstone rock sample

In this study, in the first stage, several sandstone rock outcrops were needed for zeta potential and contact angle experiments. For these experiments, the rock powders and thin slices were prepared from the sandstone rock slabs. It should be mentioned that the rock slabs were not aged in formation brine first. Also, to obtain rock powder with uniform particle size and appropriate range for measuring zeta potential, the sandstone outcrops were crushed and passed through 2 sieves with consecutive meshes (mesh numbers 325 and 400). The particle size distribution in the lower sieve is in the range of 37–44 microns^5^. X-ray diffraction fluorescence (XRF) analysis was used to evaluate the lithology of the core because the lithology of rock showed an effective impact on zeta potential measurement. The results obtained from the XRF analysis show that the mineralogy of the rock outcrop presented in Table 5. The abbreviation of LOE stands loss on ignition in Table 5.

**Table 5:** The results of XRF analysis of core samples.

Element	SiO_2_	Al_2_O_3_	BaO	CaO	Fe_2_O_3_	K_2_O	MgO	MnO	Na_2_O	P_2_O_5_	SO_3_	TiO_2_	LOI
Value (%)	36.59	2.14	<	29.54	1.65	0.45	2.22	>	0.33	0.08	0.09	0.13	26.79

### Methods

#### Brine compatibility studies and determining the most optimal injection water for preparation and making low salinity water

**Figure 2:**
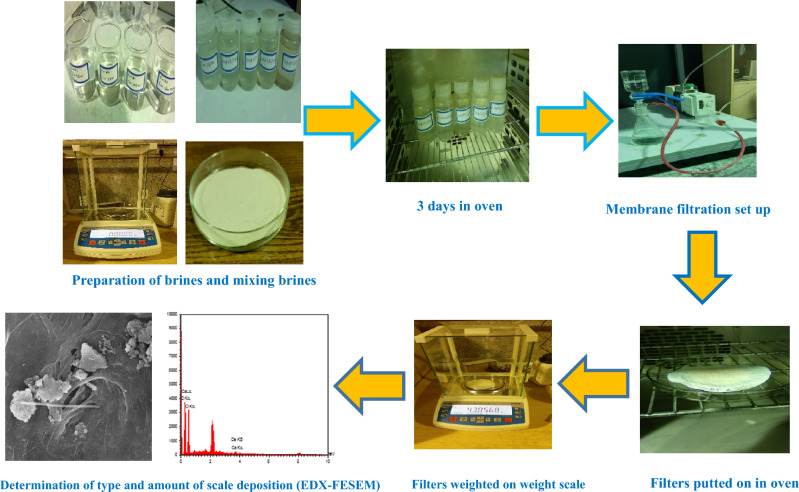
A schematic diagram of compatibility tests and determination of the type and amount scales.

In addition to the thermodynamic OLI ScaleChem software according to temperature, pressure, and pH changes, also laboratory experiments were performed to judge the compatibility/incompatibility of formation and injection waters to determine the mass and types of scale precipitation. Laboratory compatibility tests were conducted to determine the scale precipitation of mixing formation water with injection water and selecting the best injection water for the preparation of the optimum concentration of low salinity water. A schematic diagram of compatibility tests and determination of the type and amount scales is shown in Fig. 2. Compatibility tests were performed at atmospheric pressure. To assess the impact of the salinity of the injection water on the mineral scale deposition, injection waters with different types of salinity including without dilute, 2 (SW#2D), 5 (SW#5D), 10 (SW#10D), 15(SW#15D), 20 (SW#15D) and 25 (SW#25 D) times diluted seawater (SW) were synthesized. Table 6 shows the compositions used to make up injection brines for the initial compatibility test and determine the optimal injection brine with the lowest mineral scale deposition. Ultimately, low salinity waters were prepared based on this optimal water composition. Afterward, injection water samples (including SW, SW#2D, SW#5D, SW#10D, SW#15D, SW#20D, and SW#25D) were mixed with formation brine at different mixing ratios of 20%, 40%, 50%, 60%, and 80%. To perform each compatibility test, 50 cm^3^ of each injection brine was filtered, poured into an autoclavable bottle, and mixed with 50 cm^3^ of formation water. The autoclavable bottles were placed in an oven at atmospheric pressure and reservoir temperature (65 °C) for 3 days for the effective reaction between ions. Also, these mixed waters were shaken twice in the morning and evening. The main cause of scale formation is the supersaturation of a solution of one or more salts in the aqueous phase^36,52,56^. After 72 h, all samples were passed through a 0.22 µm membrane filter, and then the mass of scales on the membrane filter was calculated by an electronic top pan balance. In addition, the filters were exposed to deionized water by filtering deionized water to remove the residual NaCl (halite) salt, which probably covered the scale's surface because NaCl salt was a soluble salt in water and its deposit was unimportant.

**Table 6:** Different low-salinity water compositions of Persian Gulf seawater with types of dilution.

Ions	Unit	Seawater (PG)	SW#2D	SW#5D	SW#10D	SW#15D	SW#20D	SW#25D
Na^+^	mg/l	126,53	6326.50	2530.60	1265.30	843.53	632.65	50.52
K^+^	mg/l	420	210	84	42	28	21	16.8
Ca^2+^	mg/l	498	249	99.60	49.80	33.20	24.90	19.92
Mg^2+^	mg/l	1408	704	281.6	140.8	93.87	70.4	56.32
SO_4_^2−^	mg/l	3037	1518.50	607.40	303.70	202.47	151.85	121.48
Cl^−^	mg/l	22,598	11,299	4519.6	2259.8	1506.30	1129.90	903.92
HCO_3_^−^	mg/l	73	36.50	14.60	7.30	4.87	3.65	2.92
TDS	mg/l	40,687	20,343.50	8137.40	4068.70	2712.47	2034.35	1627.48
Salinity	mg/l as NaCl	37,173.71	18,586.85	7434.74	3717.37	2478.24	1858.68	1486.94
pH	–	8.138	8.13	8.13	8.13	8.13	8.13	8.13
EC	ms/cm at 25 °C	58	29	11.60	5.80	3.87	2.90	2.30

#### Wettability alteration process

**Figure 3:**
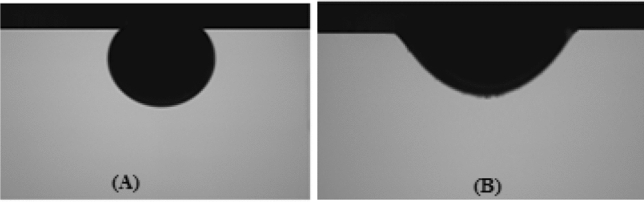
Images of contact angle change of an oil droplet on the sandstone surface solutions before (**A**) and after (**B**) wettability alteration aged by 0.01 M stearic acid and n-Heptane.

According to liter review^57–64^, fatty acids could change the surface rock wettability from water-wet to oil-wet. As the surface of sandstone rocks was usually water-wet due to chemical structure, the initial wettability of rocks was altered toward oil-wet for the test, using a stearic acid/n-heptane mixture. Stearic acid is a fatty acid with a chemical formula of CH_3_ (CH_2_)16COOH and a molecular mass of 284.48 g/mol and is soluble in normal heptane and heavier alkanes. Normal heptane at ambient temperature has viscosity and density of 0.879 cp and 0.73 g/cm^3^, respectively. The sandstone rock samples were oil wetted by 0.01 molarity of stearic acid/n-heptane liquid at 65 °C for 4 days. Figure 3 shows the wettability alteration situation of thin slices before and after the aging process. The contact angle experiment (Fig. 3B) proved the wettability shift toward oil wetness.

#### Field emission scanning electron microscopy/energy dispersive X‐ray spectrometry (FESEM/EDX)

In this study, FESEM-EDX was performed on the post-treatment filters to examine the potential scale deposition after the interaction of different injection brines with the formation brine. FESEM images could help to determine the local scaling behavior and surface modification effect of the treatment brines. In addition, EDX results were applied for the local elemental analysis and chemical characterization of the post-treatment filter surface. Also, FESEM images were interpreted utilizing the results from the contact-angle measurement subsection, which could provide a qualitative investigation of the wetting aspects of the rock surfaces. The surface morphology of filters was investigated with FESEM images utilizing Tescan MIRA3 FEG (Czech Republic) with an acceleration voltage of 20 kV. In addition, Tescan MIRA3 was used for EDX analysis to identify the number of atoms filled on the surface of filter samples.

#### Apparatus for measuring the zeta potentials

Zeta potential (Zp) can measure the injected water samples based on EOR performance^65^. Zp presents the value of the surface charge of the particles in a colloidal solution. Electrophoresis is defined the move of a charged particle proportion to the solution that is suspended under the effect of a utilized electric field^66^. The electrophoretic mobility of suspended sandstone particles and brine was measured by the apparatus, after the Zp value was determined. The Zp of colloidal dispersions was measured using laser Doppler electrophoresis (LDE). In the LDE, a voltage across a cell is used, and the electrophoretic mobility of particles is obtained from the frequency shift observed in the scattered light^67,68^. The electrophoretic mobility measure is the best and simplest manner to estimate the Zp^69^. Finally, Zp is dependent on electrophoretic mobility and calculated using the Henry equation as follows^66,70^:

4 $$ {\text{U}}_{{\text{E}}} { = }\frac{{{2}\varepsilon {\text{ z F(k a)}}}}{{{3}\eta }}, $$

where, U_E_ is electrophoretic mobility, z is zeta potential, $\varepsilon$ is dielectric constant, F (ka) is Henry’s Function, and $\eta$ is viscosity. The changing composition of injection brines and diluted seawater levels also change the surface charge of the surface rock. The changes in surface charges show a good sign of injection brine success to wettability modification of surface rock toward water-wet^39^. The surface charge of solutions was measured by applying the Malvern Zetasizer Nano ZS apparatus for oil-wet sandstone particles scattered in the desired aqueous solution. The amount of surface charge was reported as the Zp of different brine samples. As above mentioned, the rock powders were aged in n-Heptane and 0.01 M stearic acid at 65 °C and 101.3 kPa pressure until rock powders became oil-wet. The aged sandstone particles scattered in the water sample were prepared by mixing 0.5 g of powdered sandstone particles with 50 cm^3^ of brine, representing by weight 1% of an aqueous suspension^45^. The solutions were sonicated for 20 min using a sonication tool and held for 48 h to reach the equilibrium conditions. An agreeable Zp amount was chosen about the mean amount from each sample.

## Results and discussion

### Identification and determination of scales using commercial OLI ScaleChem software

**Figure 4:**
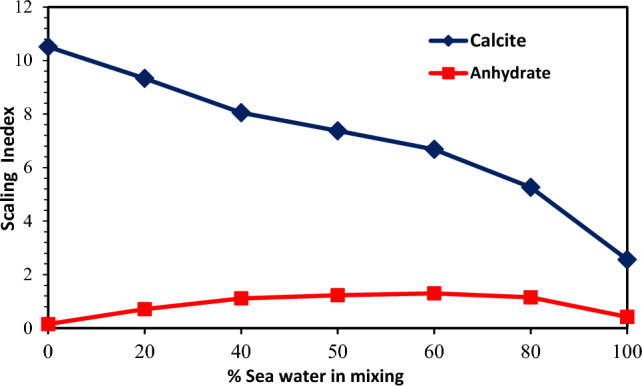
The scaling index of CaCO_3_ and CaSO_4_ scales at different mixing ratios of injection water (seawater) and formation water.

**Figure 5:**
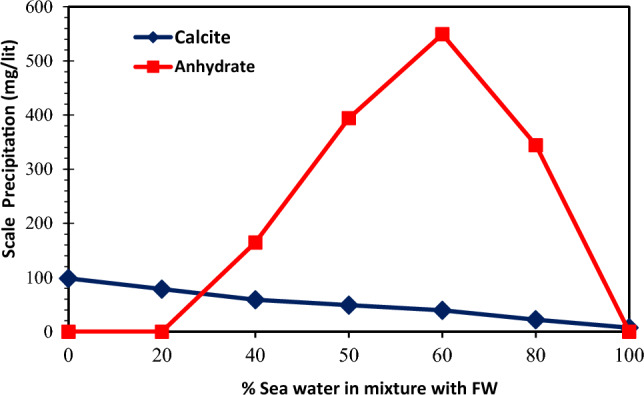
The amount of scale precipitation of CaCO_3_ and CaSO_4_ scales at different mixing ratios of injection water (seawater) and formation water.

As previously mentioned, the most important factor on scale formation in an aqueous solution is the supersaturation condition. This condition occurs when the concentration of dissolved salts in an aqueous solution is higher than a saturated solution^12,36,52^. Therefore, a supersaturation situation leads to precipitation, deposition, and the crystal growth of carbonate scales. For each mix of two incompatible waters (one including the excessive values of bicarbonate ions and the other including the excessive values of calcium ions), the Scaling Tendency (ST) is defined as the ratio of the ion activity product (IAP) divided by the equilibrium constant (Ksp). Consequently, it is the stimulation force for the precipitation reaction^36^. A greater degree of supersaturation can result in a greater possibility for CaCO_3_ precipitation. Mixing seawater (including high bicarbonate ions) with formation brine (including high calcium ions) led to calcium carbonate precipitation. Before performing laboratory tests designed to study the compatibility of brine, the OLI Scale software was applied to predict the potential of mineral scale precipitation due to mixing the injected seawater and low-salinity brines with formation water (see brine composition analysis data in Tables 2 and 6)^71^. It could help us describe different types of mineral scales formed in the aqueous solutions after mixing different low salinity brines with FW^35^. In addition, the simulation can determine the mixing ratio that the potential of scale formation occurs at the highest value. The injection brines and FW's chemical ionic composition were used in the mixed brine simulation model to estimate mass precipitation rates and the types of scales. The prediction of CaCO_3_ and CaSO_4_ scale tendencies because of mixing the injected seawater with formation water is presented in Figs. 4 and 5, respectively. The maximum precipitation was 549.18 mg/L for CaSO_4_ at 60% seawater and 78.65 mg/L for CaCO_3_ at 20% seawater. The ionic activity and the solubility are important factors on scale deposition^12^. Therefore, the reason of maximum scale formation at mixing ratio of 60% was that by increasing the mixing ratio of seawater to 60%, the concentration of bicarbonate, calcium, and sulfate ions increased to the same ratio. Consequently, in this condition, both the ionic activity and the solubility increased. In other words, by increasing the percentage of seawater in mixing brine up to 60% that the interaction between anions and cations was increased and the solubility of mineral scales in brine was reduced. Therefore, there was a maximum mixing ratio in which the maximum amount of scale cannot dissolve in solution and the superstation conditions increased. However, after this mixing ratio, as the mixing ratio became more increased, the activity of calcium and sulfate ions decreased, and the solubility of mineral scales in brine increased^12^. As a result, due to the reduction of superstation conditions, the amount of total scale deposition decreased. After changing the composition of seawater as low salinity water at different diluted seawater levels, the value of CaSO_4_ precipitation was insignificant compared with that of CaCO_3_ as the increasing dilution and reduction of the salinity seawater.

To obtain more accurate results, the impact of salinity on scale precipitation and the impact of the salinity of the injection SW on the value of CaCO_3_ precipitation were investigated. Accordingly, optimum composition and salinity with increasing dilution and different salinities of injected SW were determined. Thus, the mixing proportion of seawater and formation brine showed no effect on the outcomes. The 50% mixing proportion of low salinity water with a different degree of dilution mixed with formation brine was chosen to conduct the compatibility tests.

### The effect of salinity and dilution time of SW at different mixing ratios on the scale deposition

**Figure 6:**
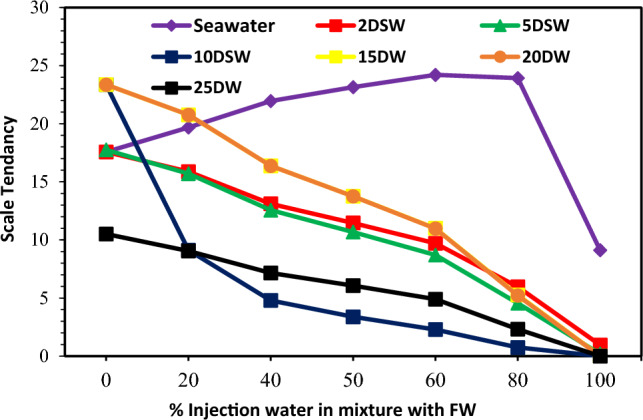
CaCO_3_ (Calcite) scale tendency at reservoir conditions.

**Figure 7:**
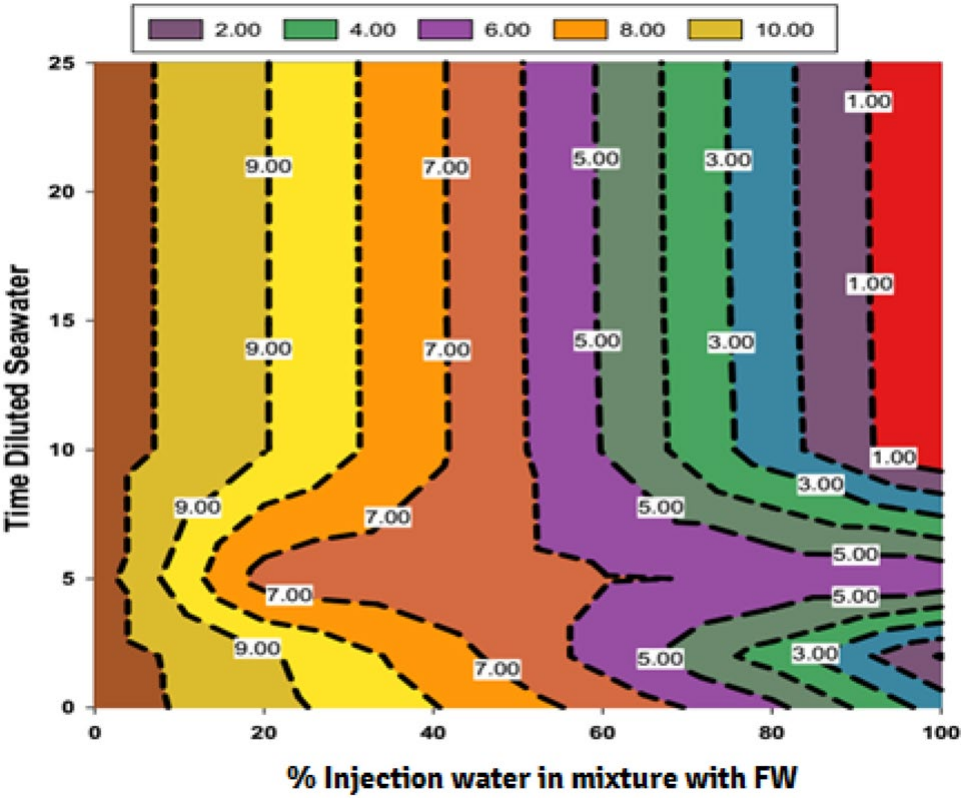
Counter plot of saturation ratio (SR) for diluted seawater ratios versus mixing ratios.

**Figure 8:**
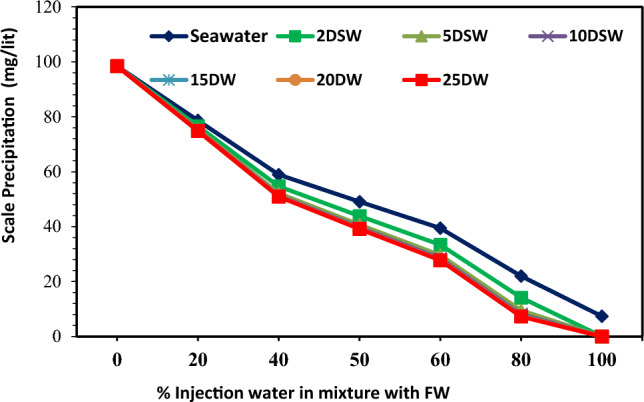
CaCO_3_ scale precipitation at reservoir conditions.

**Figure 9:**
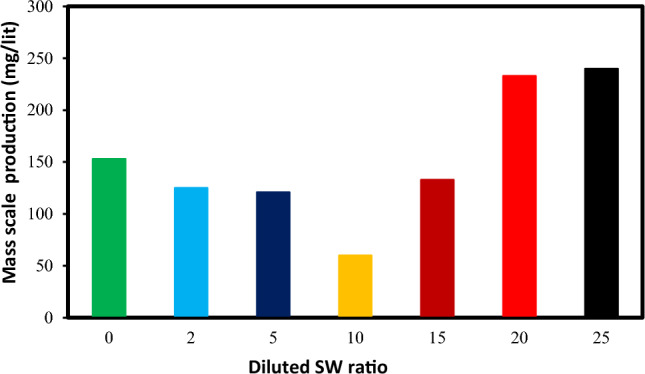
The amount of scale precipitation of mixing the diluted seawater with formation water (FW: SW # 0, 2, 5, 10, 15, 20, and 25 D).

**Figure 10:**
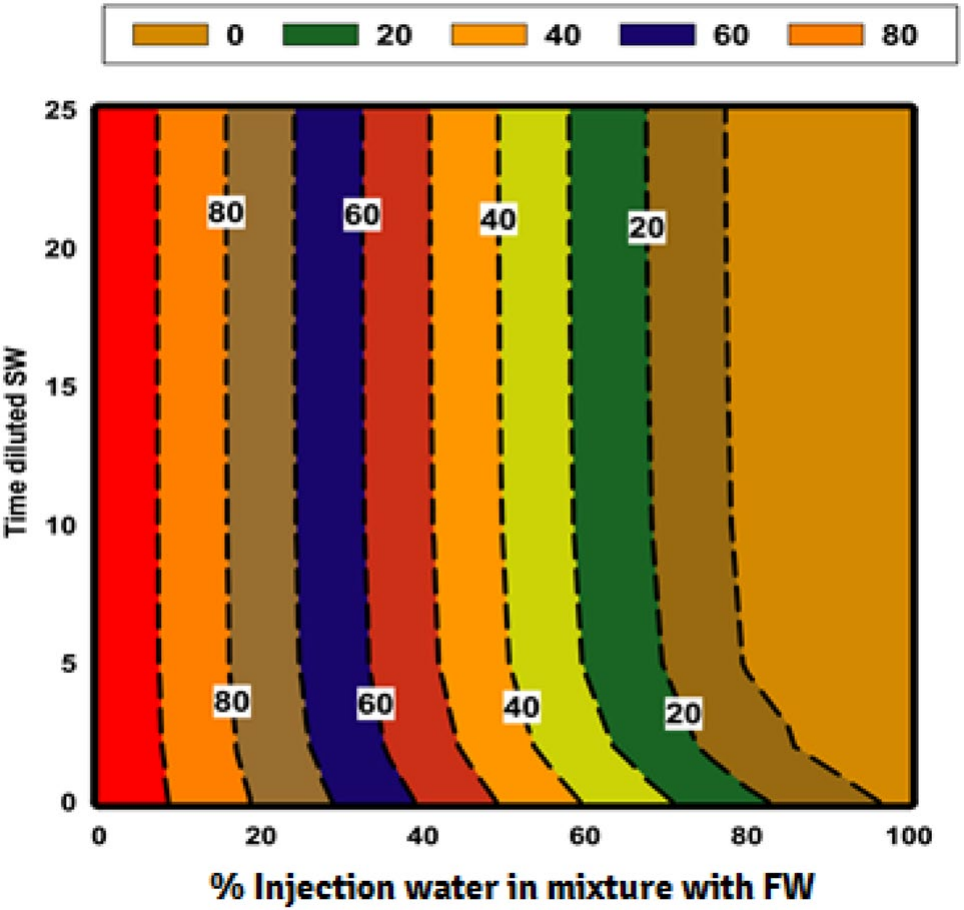
Scale deposition counters for different mixing ratios and different injection low salinity brines.

**Figure 11:**
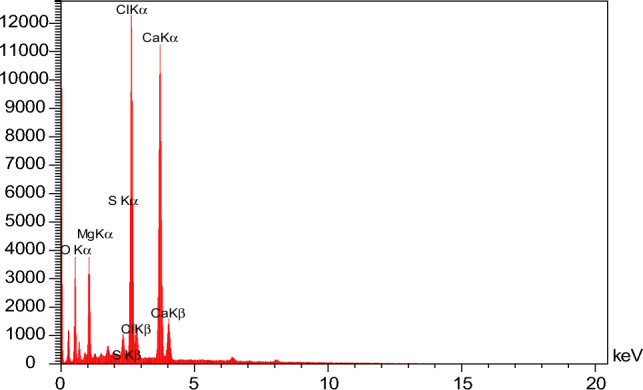
EDX analyses formation water of Table 7.

**Figure 12:**
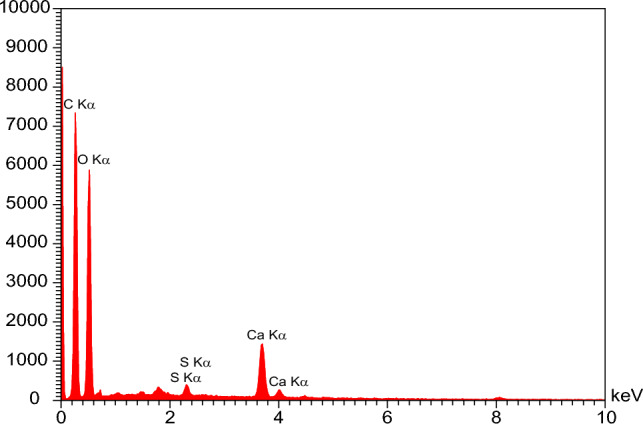
EDX analysis of seawater and formation brine composition at 50% mixing ratio of Table 8.

**Figure 13:**
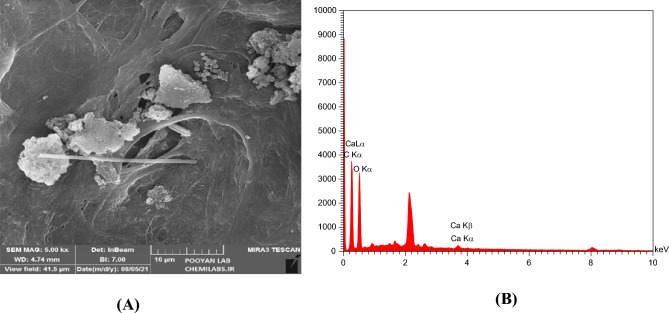
(**A**) FESEM image and (**B**) EDX analysis of mixing of LSW (SW#10D) and formation brine at 50% mixing ratio.

The CaCO_3_ mineral scale tendency and scale deposition result in various mixing ratios of seawater and low salinity water in different diluted seawater levels and formation brine are presented in Figs. 6, 7 and 8, respectively. Tables 2 and 6 shown that some Ca^2+^ and HCO_3_^−^ ions were observed in both formation brine and injected low salinity water can be formed CaCO_3_ scale. The value of the mineral scale was estimated using the OLI ScaleChem and outlined in Fig. 6 for various dilutions and salinities of SW at different mixing ratios of SW/FW. Figure 7 shows the distribution of saturation ratio magnitude contours. Figures 6 and 7 shown that the amount of the CaCO_3_ scale tendency was higher than 1; therefore, the CaCO_3_ scale was formed at different mixing ratios of SW and FW. As depicted in Figs. 6, 7 and 8, by increasing the mixing ratio from the injected waters to the oil reservoir, the amount of CaCO_3_ precipitation decreased. Also, as seawater's salinity decreased and dilutions increased, the ST and scale precipitation decreased due to calcium carbonate. Figures 6, 7, 8, 9 and 10 depict that the ST of 10-time diluted seawater (SW#10D) has a more noticeable decrease than other injected smart water, and its curve shows a greater decreasing trend. According to Fig. 7, the amount of scale deposition after 10-time diluted seawater (SW#10D) does not much change with increasing dilution to 15, 20, and 25 times. According to this figure, increasing dilution more than 10 times is not reasonable and cost-effective on a field scale in terms of cost and injecting a large volume of diluted injection water into the oil reservoir. Therefore, the optimal low-salinity injection water was 10 times diluted seawater. The laboratory results obtained from the compatibility test in a mixing ratio of 50:50 percent of SW/FW show that the lowest mineral scale formation rate occurred in 10 times diluted seawater. Figure 9 shows the amount of scale deposition of SW/FW in possibility and the actual situation in porous media of oil reservoir as mixing ratio of 50:50 percent of both brines. According to the compatibility test results and Fig. 8, the 10 times diluted SW (SW#10D) of Persian Gulf seawater composition has the highest solubility of salts and the least amount of mineral scale precipitation with the mixing of formation water. Therefore, the low-salinity water composition was the optimal composition of injected water into the reservoir. Also, the compatibility test results were consistent with the software simulation results. The scale amount in brine is related to total salinity (TDS) and reactive ion concentrations^12^. Prior to SW#10 composition (OLSW), the further dilution ratio, the concentration of reactive ions was reduced. Therefore, the ionic activity and the solubility product parameters reduce, and the salinity parameter is a prevailing factor over the reactive ion concentration, and finally the total mass scale precipitation reduces. After that, the further dilution times further than SW#10 composition (OLSW), the activity of reactive ions (Ca^2+^and HCO_3_^−^) increases in spite of the reduction in salinity of the brine. This means that the active ion amounts prevails over the salinity parameter, and this causes rising the activity of Ca^2+^and HCO_3_^−^ ions and arrives to quicker reactivity rise between the anionic and cationic ions, and finally higher scale deposition can occur^12^. At optimal salinity and brine composition of 10 time-diluted seawater (SW#10D), there was an equilibrium state between ions concentration, pH, electrical conductivity, ionic strength, and the amount of carbon dioxide solution in the water. After this optimum condition, due to the reduction in the ions’ activity in the complex formation, the amount of scale precipitation decreased, and the mineral scale was formed slowly or stopped. Therefore, increasing the dilution without considering the active ions and their activity cannot guarantee reducing scale deposition during low salinity water flooding under reservoir conditions. After completion of compatibility tests, SEM–EDX analysis was conducted on scales deposited on filters owing to the mixing of formation brine and different injection brines to detect the kind and configuration of different scales. Also, the precise diagnosis of scale species and elemental composition was presented via EDX^45^. Table 7 shows EDX analysis of formation water. Also, Fig. 11 shows the EDX spectra for formation brine in Table 7. EDX analyses of formation brine are shown in Fig. 11 and Table 7 are proved that Ca, O and C, S atoms have created small amounts of sulfate carbonate scale, and the prevailing type of scale is calcite. Table 8 shows EDX analysis of the 50% mixing ratio of seawater and formation water. According to EDX analysis, small amounts of sulfate scale and carbonate scale can be formed and the prevailing type of scale is calcite (Table 8). The EDX spectra also proved the low amount of CaSO_4_ in the 50% mixing ratio of injection water and FW. EDX of mixing brines are shown in Fig. 12 and Table 8 that Ca, O and C atoms have created the prevailing type of calcite scale. However, lower amounts of S atom along with Ca, C, and O in the mixing composition indicate that a small amount of calcium sulfate can be formed, which is removed over time by diluting seawater due to a decrease in SO_4_^2−^ concentration. Figure 13A,B show a FESEM image with a zoom of 5000 times and the EDX spectra of 50% LSW and formation water, respectively. In addition, EDX analysis of this composition is proved amounts of calcium carbonate scale (Table 9). The EDX spectra also proved the low amount of CaCO_3_ in the 50% mixing ratio of OLSW (SW#10D) and FW (see Fig. 13B). As can be seen in Fig. 13B, in 50% injected brine and formation water composition, atomic picks of Ca, C, and O atoms are greater, which illustrates the scale deposition of CaCO_3_. The same findings can be deduced from Fig. 12 for other samples. FESEM-EDX of mixing brines are shown in Fig. 13 and Table 9 that divalent cation (Ca^2+^) had created the prevailing type of scale that is calcite.

**Table 7:** EDX analysis of formation water.

Elt	W%	A%
C	10.27	19.26
O	32.85	46.23
Mg	1.03	0.95
Cl	25.49	16.19
Ca	28.11	15.79
S	2.25	1.58
Dominant scale	CaCO_3_ and CaSO_4_

**Table 8:** EDX analysis of mixing ratio 50% of formation water-seawater.

Elt	W%	A%
C	37.06	46.07
O	54.02	50.42
Ca	1.90	0.89
S	7.02	2.62
Dominant scale	CaCO_3_ and CaSO_4_

**Table 9:** EDX analysis of mixing ratio 50% of formation water-SW#10D in SEM image of Fig. 13.

Elt	W%	A%
C	35.36	42.76
O	61.96	56.27
Ca	2.68	0.97
Dominant scale	CaCO_3_	

### Effect of concentrations of ions of injection brines at different mixing ratios and dilution ratios on CaCO_3_ scale precipitation

**Figure 14:**
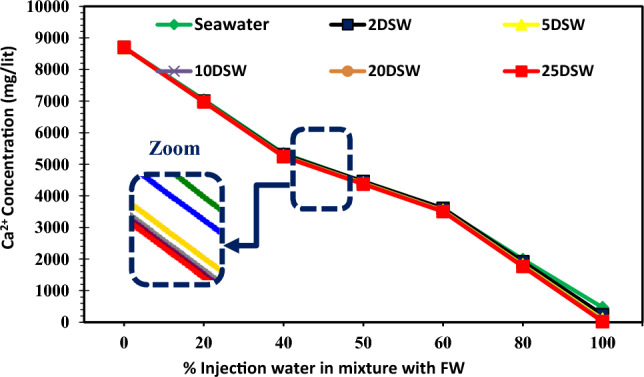
Variation of Ca^2+^ ion at different mixing ratios of injected water in diluted seawater level with the formation brine.

**Figure 15:**
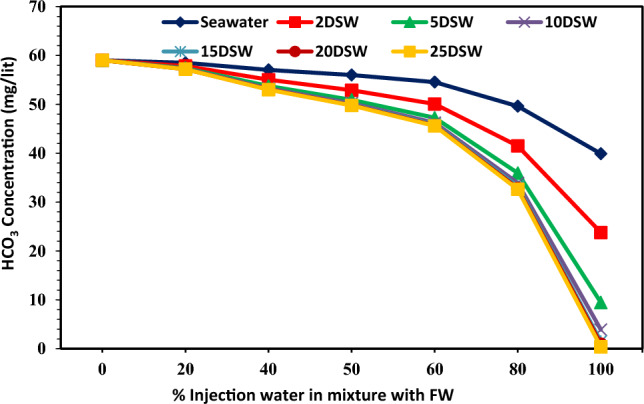
Variation of HCO_3_^−^ ion at different mixing ratios of injection water in diluted seawater level with the formation brine.

**Figure 16:**
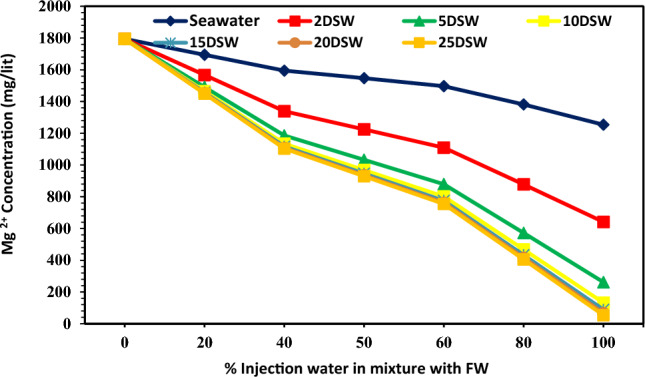
Variation of Mg^2+^ ion at different mixing ratios of injection water in diluted seawater level with the formation brine.

Table 2 shows that the concentration of bicarbonate ion (HCO_3_^−^) in seawater is very small (73 mg/L), whereas it is present in the formation water in large quantities (293 mg/L). Therefore, it can cause many problems due to the deposition of calcium carbonate in the production operating systems of this oil reservoir. Figure 14 shows Ca^2+^ ion concentration in different injection SW and low salinity waters versus different mixing ratios of SW/FW. The amount of calcium concentration has a declining trend with increasing dilution of seawater, and almost the amount of trend changes is constant with increasing mixing ratios. Also, the trend of Ca^2+^ concentration is almost within close range at different mixing ratios of SW/FW. The amount of Ca^2+^ ion concentration does not change by increasing the dilution of 10 times diluted seawater; therefore, these results correspond to the laboratory and simulation findings. The amount of HCO_3_^−^ ions was decreased by increasing mixing ratios and salinities (Fig. 15). As can be seen in Fig. 15, 10 times diluted seawater was an optimum concentration of bicarbonate ion; after this salinity, no change was observed in the trend of decline curve. In other words, the effect of optimal salinity is a complementary effect due to changes in calcium and bicarbonate ions, and the detection of optimum salinity can improve by changes in Mg^2+^ and HCO_3_^−^ concentrations prior to Ca^2+^ concentrations. Reducing the concentration of bicarbonate ions to 10 times the initial amount in seawater, the amount of scale formed decreases (Fig. 15), and after this optimum LSW the amount of scale deposition does not change more. Therefore, diluting seawater more than 10 times is not economically suitable. Due to high charge density, Mg^2+^ ions are well covered at low temperatures by water molecules compared to Ca^2+^ ions. But, at high temperatures (above 70 °C) due to dehydration, its activity increases. Therefore, Ca^2+^ ions' activity in the solution is overshadowed and reduces the reaction of SO_4_^−^ ions with Ca^2+^. As the solubility of calcium sulfate increases, less scale will be formed^12^. Mg^2+^ ions can inhibit the rate of calcite nucleation. The concentration of the new nuclei is too dilute for each additional interference. The subsequent nuclei are then free of Mg^2+^ inhibition and have a normal growth rate, repressing the identity of reaction^72^. It can be expected that with increasing the concentration of magnesium ion, the amount of calcium carbonate precipitation corresponding to the rate of dilution ratio decreased (Fig. 16). However, the effect of Mg^2+^ ion has an intensity in low salinity brines. The addition of Mg^2+^ ions during calcium carbonate scale formation considerably affects the apparent solubility of the magnesium calcite phase formed. Therefore, effective scale inhibitors by magnesium ion may be due to active adsorption sites and a reduction in the thermodynamics of the formation of magnesium calcite scales^73^.

### Effect of pH and CO_2_ concentration of SW at different salinities on scale formation

**Figure 17:**
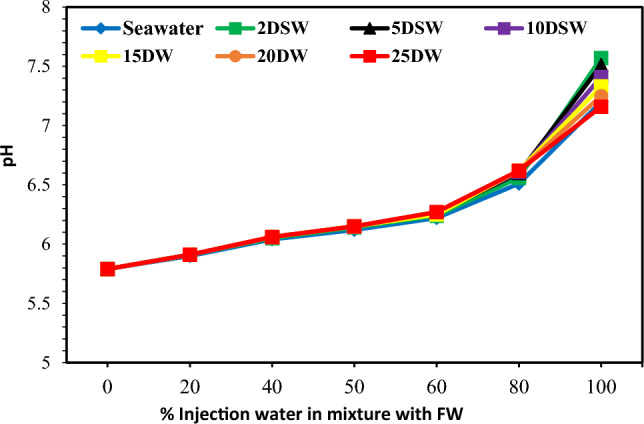
Variation of pH value at different mixing ratios of injection water in diluted seawater level with the formation brine.

**Figure 18:**
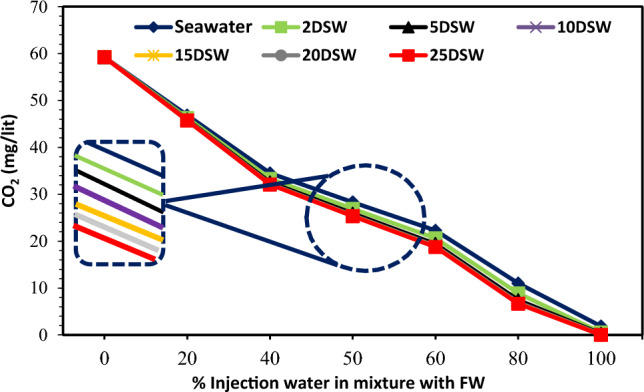
Variation of CO_2_ concentration at different mixing ratios of injection water in diluted seawater level with the formation brine.

The mechanism of calcium carbonate scale formation with sulfate scales is different. This scale is mainly formed in oil reservoirs because of CO_2_ emissions stemming from bicarbonate ions, resulting in a sudden decrease in pressure. The amount of CO_2_ in the solution affects the pH of water and the solubility of CaCO_3_. At low pH values, the rate of calcium carbonate deposition is lower, and conversely, at high pH values, this amount is higher^47^. When CO_2_ is removed from the solution, the pH increases and the solubility of the soluble carbonates decreases, and most of the soluble bicarbonates become insoluble and also in the form of insoluble carbonates. Therefore, the solubility of bicarbonate ions at ambient pressure is very low. But, even this small amount can cause scale deposition when mixing seawater with the formation water. So, in Fig. 18 is observed that with increasing the rate of water injection mixed with the formation water, the amount of dissolved carbon dioxide decreases. Also, it shows that the downward trend in the amount of carbon dioxide after 10 times diluting with water is almost constant. The results are fully compatible with the results of changes in bicarbonate ions in Fig. 15. Figure 17 shows pH changes with an increasing percentage of mixing of two brines. Therefore, with increasing the percentage of mixing of two brines, the pH increases. As previously mentioned, when CO_2_ is released from the solution, the pH of the solution increases (Fig. 18).

### Effect of ionic strength and conductivity at different mixing ratios on CaCO_3_ scale precipitation

The ionic strength (IS) of a solution depends on the concentration of ions amount of an aqueous solution. Therefore, the solution's ionic strength is considered a function of the concentration of total ions recognized in the solution. The IS of an aqueous solution can be defined as follows^74^:

5 $$ {\text{I}} = 1/2\Sigma {\text{C}}_{{\text{i}}} {\text{Z}}_{{\text{i}}}^{2} . $$

**Figure 19:**
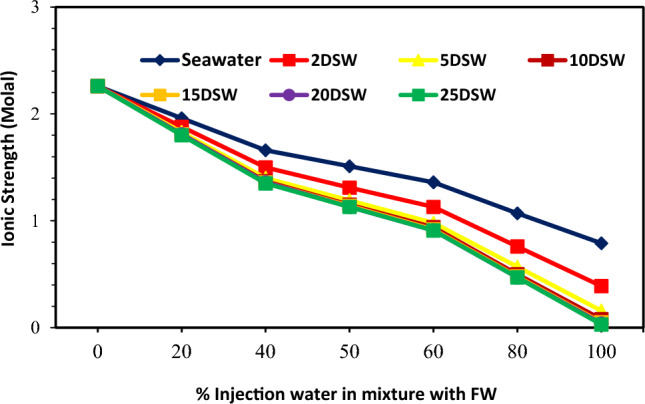
Variation of ionic strength at different mixing ratios of water injection in diluted seawater level with the formation brine.

**Figure 20:**
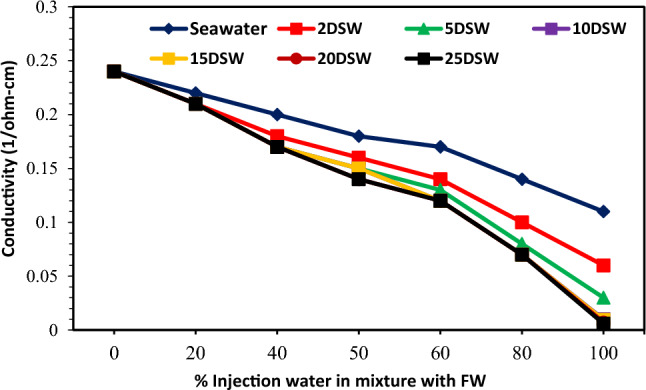
Variation of conductivity at different mixing ratios of water injection in diluted seawater level with the formation brine.

C_i_ represents the concentration of ion *i* (M = mol/L); Z_i_ denotes the charge number of ions, and all ions in the solution are represented by the sum^74^. The solubility product (K_sp)_ is also expressed in molality (m) concentrations and depends on both temperature and IS^36^. The solubility of the CaCO3 is highly influenced by the concentration of other ions observed in the system like Mg^2+^^72^. The solubility value of the CaSO_4_ is higher than the SrSO_4_, which in turn is nearly one and one-half orders of the amount higher than that of the BaSO_4_^75^. The variations of IS at different mixing ratios of injection brine of diluted seawater levels with formation brine are shown in Fig. 19; with increasing dilution times of seawater and decreasing SW salinity, IS decreased at different mixing ratios. After 10 times diluted seawater, the IS amount has not changed, and it is almost constant. In addition, according to Figs. 6, 7, 9, and 19, the ST of calcium carbonate was highly influenced by both the concentration of other ions observed in the system and the ionic strength. Then, as previously shown, after 10 times diluted seawater, the amount of activity ions’ concentration at different mixing ratios of SW/FW was nearly constant. Therefore, it is expected that IS for other low salinity waters such as 15, 20, and 25 times diluted seawater has no change. One simple way to determine the concentration of suspended minerals in water is to measure EC. Distilled water or pure water is almost not a conductor of electricity. However, if salts are present in water, water can be a conductor of electricity. Therefore, the higher amount of salts dissolved in water, the higher EC; in other words, its electrical resistance decreases. The conductivity of water indicates ability of the electrification current to pass through the aqueous solutions. The electric current in solutions is guided by ions' motion, and the higher number of ions (the higher the concentration of soluble salts), the greater ionic mobility and the higher EC. Since EC is directly related to TDS and water-soluble salts, by measuring it, the amount of soluble solid particles and thus depositions of these in the solution can be measured by reducing the EC of solution^76^. The solution's amount of conductivity shows a suitable method for evaluating scale deposition during mixing low salinity waters as the mixing of injection water with formation water at different ratios^13^. Results of conductivity values in Fig. 20 show a decreasing trend for all brines with an increasing mixing ratio of SW/FW. Also, similar results were obtained of scale tendency and scale deposition in Figs. 6, 7 and 8. The concentration trend of activity ions for CaCO_3_ scale formation at different mixing ratios was in agreement with the results obtained from conductivity measurements of low salinity brine. The findings show an optimum composition of diluted seawater including minimum scale deposition. Reduction in seawater salinity and increasing time diluted seawater is not suitable and practicable of economically and operationally aspects at higher compositions of OLSW. Consequently, increasing the time diluted seawater without examining active ions and their activities cannot guarantee to decrease scale deposition in the low salinity water flooding.

### FESEM analysis and contact angle measurements for determination of wettability alteration

**Figure 21:**
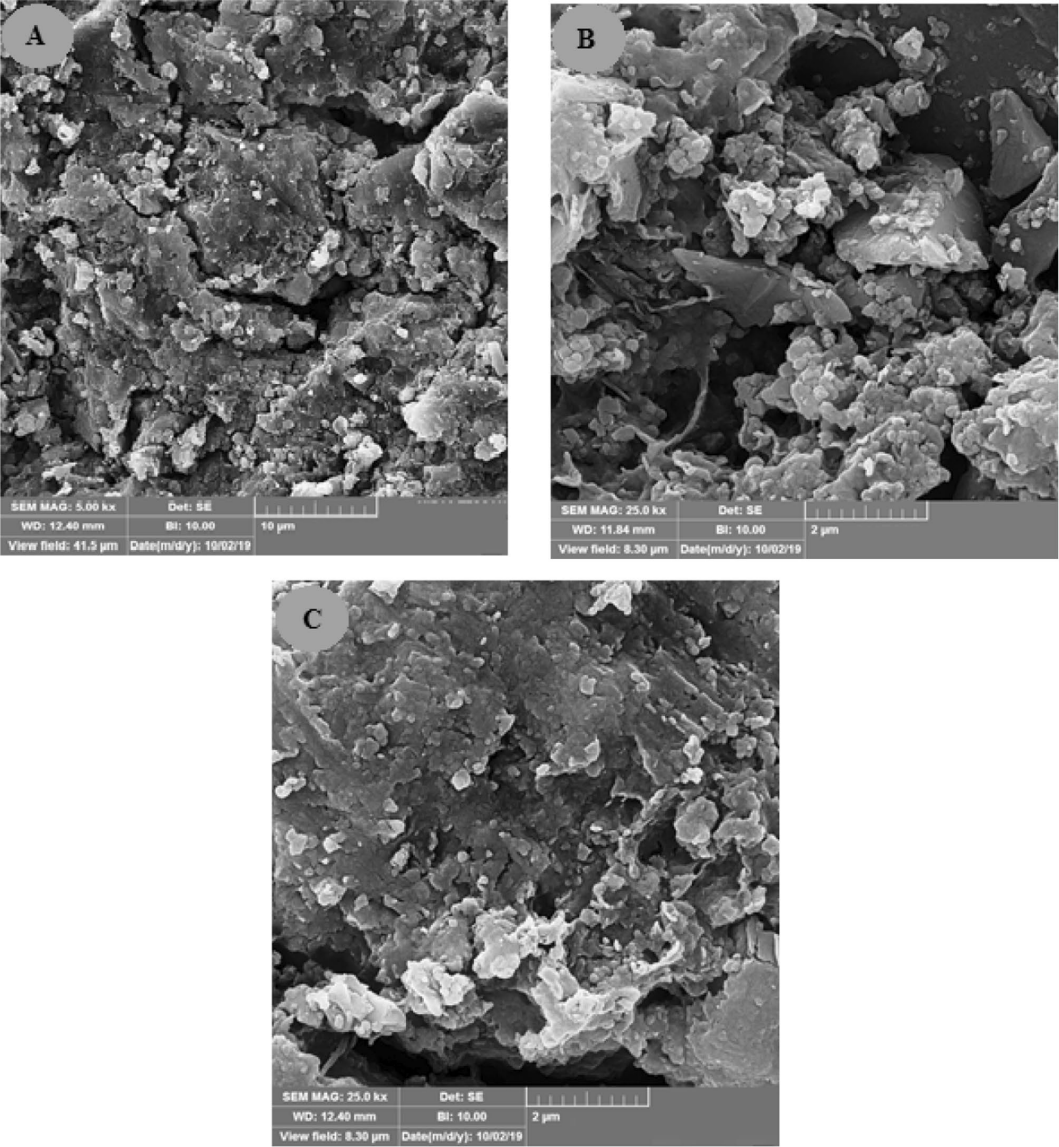
FESEM pictures of (**A**) a clean sandstone rock (water wet), (**B**) a sandstone rock aged in stearic acid and n-Heptane composition, and (**C**) an oil-wet sandstone rock aged in optimum SW#10D.

**Figure 22:**
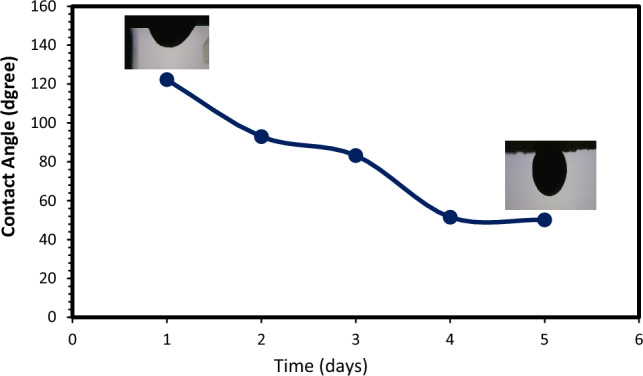
Contact angle variation of optimum low salinity water (SW#10D)/oil/sandstone rock with respect to time.

The wettability of reservoir rock has an important effective on oil recovery due to impact on liquids permeability, interaction between oil droplets and rock surface, and sticking of oil droplets on rock surface^77^. As mentioned previously before wettability alteration was one of the main mechanisms of enhance oil production by LSW and smart water flooding^78,79^. Field emission scanning electron microscopy (FESEM) can be used as a method for surface modification and the wettability alteration of carbonate and sandstone surfaces from oil-wet to water-wet^60,80–82^. The quality of changing the wettability of sandstone surface from water-wet to oil-wet, coating, and surface adsorption with optimum low salinity water (SW#10D) with minimum scale deposition was assessed using the FESEM. Figure 21 illustrates the FESEM images of surface sandstone rock before and after the low salinity water treatments. Figure 21A,B shows the sandstone plate surface before aging (water wet) and after aging (oil-wet), respectively. The following figures show that the sandstone contains particles on a smooth surface (expected crystal edge). Then, the samples were aged by fatty acid (stearic acid) and n-heptane; the smooth surface was covered with a thin layer of oil, and its morphology was significantly modified (Fig. 21B). The sandstone surface had a gibbous hill without any smooth edge because of the adsorption of crude oil on its surface. The wettability of the sandstone plate surface could change from water-wet to oil-wet. The sandstone surface was aged in optimum low salinity water (SW#10D) for 24 h. Figure 21C shows the FESEM images of the sandstone rock surface after aging. It should be noted that in an oil-wet porous media, the impact of formation water on wettability alteration is less effective^83^. The FESEM images show low salinity water well spear and adsorption on quartz surfaces. Mechanistically, negatively charged low salinity water composition can further alter the surface charge of quartz surface (Fig. 21). According to Fig. 21 and Zp results of Fig. 24, it can be concluded that the optimum low salinity water with minimum scaling alters the rock surface toward a negative charge. As a result, it could effectively modify the wettability of the sandstone surface to water-wet. Also, to rather study the effect of rock-OLSW interaction on ultimate oil recovery, the tendency and amount of wettability change should be examined. Therefore, a contact angle test was carried out at optimum low salinity water (SW#10D) with regard to time. Figure 22 shows the contact angle of optimum low salinity water (SW#10D)/crude oil/sandstone rock system with time, for 5 days to reach equilibrium. The contact angle measurements shown that contact angle in presence of OLSW reduced to 50.13° after 5 days. Therefore, the contact angle outcomes display the wettability alteration toward water-wetness over time. As a result, FESEM and contact angle findings confirmed Zp results, it was mean that OLSW was able to make sandstone surface more negative with diluting seawater and wettability changed from oil-wet toward water-wet.

### Impact of dilution, salinity and brine composition on rock surface charge and wettability alteration

**Figure 23:**
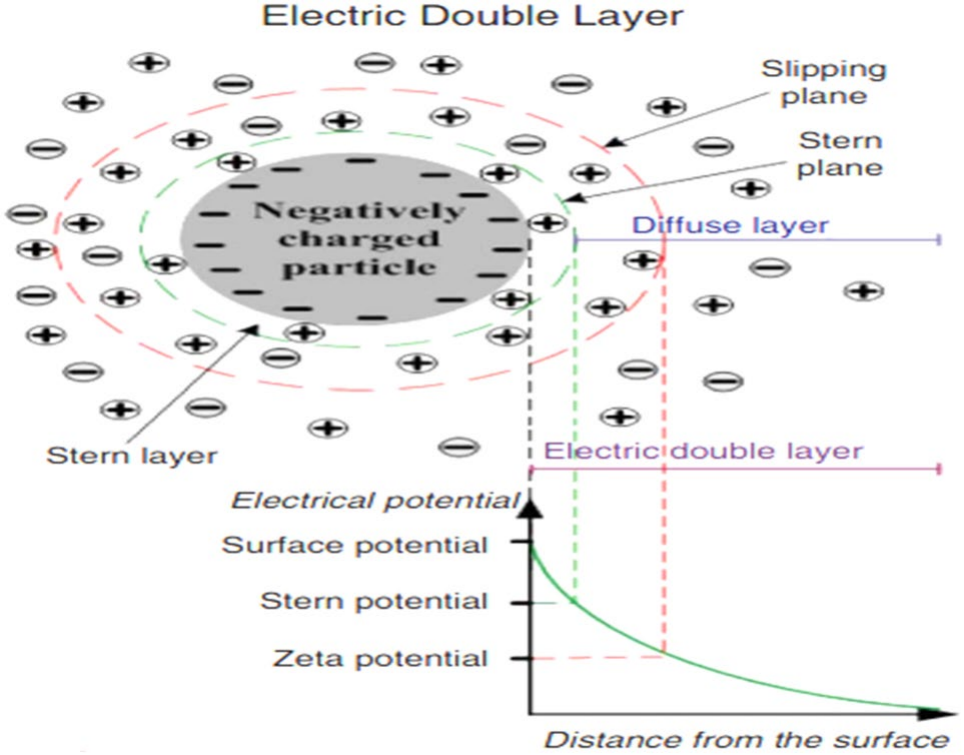
Schematic diagram of EDL in sandstone/brine system^84^.

**Figure 24:**
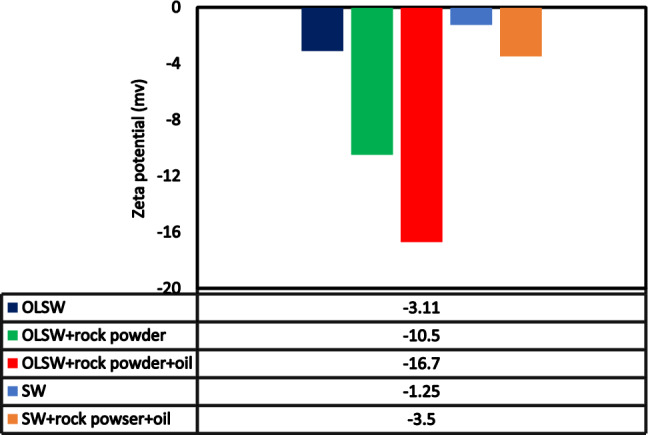
Zp values of aged sandstone rock powders scattered at different brines.

The impact of LSW is ascribed to the change in wettability, which is chiefly due to the expansion of the electrical double layer (EDL)^84^. An electrical field that adsorbs differently charged ions is created due to the charged surface being in touch with LSW; the ordered ions create a diffuse layer of charge beyond the charged surface (stern layer). Therefore, the EDL generated by the diffuse layer and the stern layer^85^. Figure 23 shows a schematic diagram of the EDL in the brine/sandstone system. Therefore, when salinity reduces a thicker water film is created which causes expansion of the EDL by LSW brine, as a result, prepares a better possibility for oil sweep efficiency^84^. An easy and fast way to investigate surface charge changes at OLSW/rock and OLSW/oil interfaces and expansion of the EDL is via measuring Zp values^86^. The Zp value is a measurement of the electrical potential in the diffuse layer of the EDL^87^. The Zp values were used to examine the surface charge changes of aged-rock powders separated by various brines, including seawater, different LSW compositions and OLSW (SW#10D) (see Fig. 24). As a result, the Zp measurements were conducted on brine/aged-rock powder and oil/brine suspensions to illustrate the impacts of dilution, ionic composition, and salinity on the EDL. Figure 24 displays the Zp values of the aged-sandstone rock powders dispersed in brines. The Zp values were the average of three measurements for the sample. Potential determining ions including Ca^2+^, Mg^2+^, and SO_4_^2−^ can importantly influence the interactions at the LSW/rock interface that straight effects the EDL in oil reservoirs^68^. These ions are attached to the rock surface and change the rock-brine interface charge^86^. Therefore, the OLSW composition caused a more negative surface charge owing to ions adsorption on the surface rock. The negatively charged surfaces can be described by the adsorption of Ca^2+^, Mg^2+^, and SO_4_^2−^ ions at the rock surface, and the CaSO_4_^−^ and MgSO_4_^−^ complexes can create on the sandstone surface^68^. The negative surface charges on the OLSW/rock and OLSW/oil interfaces can yield electrostatic repulsion between the interfaces and donate to further positive disjoining pressure^88^. On the other hand, the sandstone surface has some active sites for potential determining ions to attach and alter the charge at the rock-brine interface. Therefore, by changing the brine salinity, the competition of the ions for binding sites and their reaction with OH^−^ and H^+^ ions in solution vary, which results in different zeta potential values also the rock surface charge can change^89^. As the OLSW had an optimum salinity and ionic composition, surface charge values altered negatively. As a result, by reducing brine salinity and ionic strength (FW > SW > SW#10D), the zeta potential presents more negative values (Fig. 24)^87^. In addition, in a solution with higher salinity or lower conductivity the EDL evolves thinner. Therefore, the ions stack up on the EDL and forbid the liberation of potential determining ions^39^. When OLSW is used as injection water due to changes at OLSW/rock and OLSW/oil interfaces, which decreases the adhesion force and attractive forces (or increase of repulsion forces) between the oil and rock surface and increases the expansion of EDL, resulting in the surface rock wettability changes to water-wet conditions^84,90^. As a result, the optimum dilution and brine composition will yield the expansion of the EDL, which means a modification in the ionic brine composition. The findings are in good agreement with the contact angle measurement and FESEM findings.

## Conclusion

The principal results of this experimental study are summarized as following:

- The minimum amount scale can be formed in an optimal salinity and dilution and LSW composition (SW#10D). After this optimal amount, the ions concentration, electrical conductivity, ionic strength, pH, and scale precipitation amount do not significantly change and are almost constant. As a result, increasing dilution time at higher optimum SW#10 is not helpful from economic and operational points of view.
- A direct relationship was observed between salinity reduction, ion strength, electrical conductivity, effective ion concentrations (Ca^2+^, Mg^2+^, and HCO_3_^−^), and surface charge variations. The results showed that in an optimal salinity and dilution (SW#10D) and after this optimal amount, the values of these parameters did not significantly change.
- The simulation results and experimental tests (FESEM-EDX and compatibility test) confirmed that the CaCO_3_ scale was the major scale when mixing injection water with formation water at different ratios. In addition, the CaCO_3_ scale essentially showed a constant value in low salinity waters because it became increasingly dependent upon thermodynamic conditions when temperature and pressure were constant in different experimental conditions.
- In the optimum LSW composition (10DSW) the rock wettability changed from oil-wet toward water-wet conditions due to an increase in dilution ratio and reduction in salinity of injected water. Consequently, SW#10D is characterized by both the control potential for scale deposition and more capacity for wettability alteration at the same time.

## Data Availability

The datasets used and/or analyzed during the current study available from the corresponding author on reasonable request.
